# A Case of Legionella pneumophila With Hearing Loss Improved by Antimicrobial Therapy

**DOI:** 10.7759/cureus.80952

**Published:** 2025-03-21

**Authors:** Mizuki Takahashi, Kanata Tonosaki, Masashi Noro, Kazuma Ota, Shigeto Oyama

**Affiliations:** 1 Department of General Medicine, Towada City Hospital, Aomori, JPN; 2 Department of Otorhinolaryngology, Towada City Hospital, Aomori, JPN

**Keywords:** bilateral sensorineural hearing loss, hearing loss, legionella infection, legionella pneumophila, pneumonia

## Abstract

*Legionella pneumophila* is known to cause various complications, but hearing loss is rarely reported. A 59-year-old woman, healthy by nature, developed a fever and cough and was hospitalized with a diagnosis of *L. pneumophila*. She had symptoms of hearing loss since admission, and examination revealed bilateral sensorineural hearing loss. However, since this did not interfere with her daily life, she was followed up. After treatment with pazufloxacin and levofloxacin for pneumonia, her hearing loss symptoms improved along with the improvement of her pneumonia. This case suggests that *L. pneumophila* may be associated with sensorineural hearing loss.

## Introduction

*Legionella pneumophila* is a gram-negative rod that grows intracellularly [1]. It is transmitted via water or aerosols containing Legionella bacteria, and patients with compromised immunity are at a high risk of developing this disease [1]. Legionella infection should be suspected based on respiratory symptoms and specific laboratory data because the organism does not stain with gram stain and does not grow in normal culture. Highly specific diagnostic tests include culture, urine Legionella antigen test, and polymerase chain reaction test [2]. Among these, the urinary antigen test is useful because it is relatively simple and rapid.

Legionella infection is mainly associated with respiratory symptoms and fever, although it can be further complicated by other neurological symptoms, such as impaired consciousness, ataxia, and confusion, and gastrointestinal symptoms, such as diarrhea [3]. However, hearing loss has almost never been reported as a complication [4].

Here, we describe a case of *L. pneumophila* complicated by sensorineural hearing loss, where both pneumonia and hearing loss improved with antimicrobial therapy alone. This complication is rare in clinical practice, but as a form of hearing loss that can be expected to improve, it is crucial to report it to aid clinicians in their decision-making regarding diagnosis and treatment.

## Case presentation

The patient is a 59-year-old female with no significant medical history who has smoked 20 cigarettes per day for 39 years. She worked as a laundry worker at a hospital. She had a fever and dry cough for seven days. Five days prior, her cough worsened, and she developed bilateral hearing loss. Four days prior, she visited a local physician and was diagnosed with an upper respiratory tract infection and placed under observation. The results showed an elevated inflammatory response, with a WBC count of 11,300/μL and a CRP level of 28.58 mg/dL; a chest X-ray showed decreased permeability in the left lung field. The patient was referred to our hospital because of acute pneumonia.

Upon visiting our hospital, she was conscious. Her vital signs were as follows: blood pressure, 197/78 mmHg; heart rate, 112 bpm; respiratory rate, 30 bpm; SpO2, 94% (room air); and temperature, 38.7°C. She had a dry cough. Auscultation revealed mild adventitious sounds in the lower left lung. She experienced difficulty hearing in both ears; however, the external auditory canal was clear. No complaints of tinnitus or dizziness were reported. No other physical abnormalities were observed.

The results of blood and urine tests are presented in Table 1. The blood test showed a WBC count of 10,000/μL and an elevated neutrophil percentage of 85.8%. Biochemical tests showed elevated aspartate aminotransferase (117 IU/L), alanine aminotransferase (86 IU/L), gamma-glutamyl transferase (109 IU/L), CRP (34.2 mg/dL), and procalcitonin (1.48 ng/dL), and decreased sodium (132 mEq/L), chloride (95 mEq/L), albumin (2.8 g/dL), and total protein (6.5 g/dL) levels. There were no symptoms of sputum, so sputum culture could not be submitted. Therefore, bacterial susceptibility could not be evaluated. Blood and urine cultures were negative.

**Table 1:** Results of the blood test on admission. T-Bil: Total Bilirubin; AST: Aspartate Aminotransferase; ALT: Alanine Aminotransferase; LDH: Lactate Dehydrogenase; GGT: Gamma-Glutamyl Transferase ; Alb: Albumin; TP: Total protein; Na: Sodium; K: Potassium; Cl: Chloride; BUN: Blood Urea Nitrogen; PCT: Procalcitonin; Hb: Hemoglobin; Plt: Platelet count; Neutro: Neutrophils; APTT: Activated Partial Thromboplastin Time.

Parameter	Value	Unit
T-Bil	0.59	mg/dL
AST	117	IU/L
ALT	86	IU/L
LDH	559	IU/L
γ-GTP	109	IU/L
Alb	2.8	g/dL
TP	6.5	g/dL
Na	132	mEq/L
K	3.4	mEq/L
Cl	95	mEq/L
BUN	8.8	mg/dL
Cr	0.68	mg/dL
CRP	34.2	mg/dL
PCT	1.48	ng/mL
WBC	10000	/μL
RBC	4.84 × 10⁶	/μL
Hb	14.7	g/dL
Ht	41.4	%
Plt	2.45 × 10⁵	/μL
Neutro	85.8	%
PT	12.1 × 10³	/μL
APTT	30.5	sec
D-dimer	6.4	μg/mL

**Figure 1:**
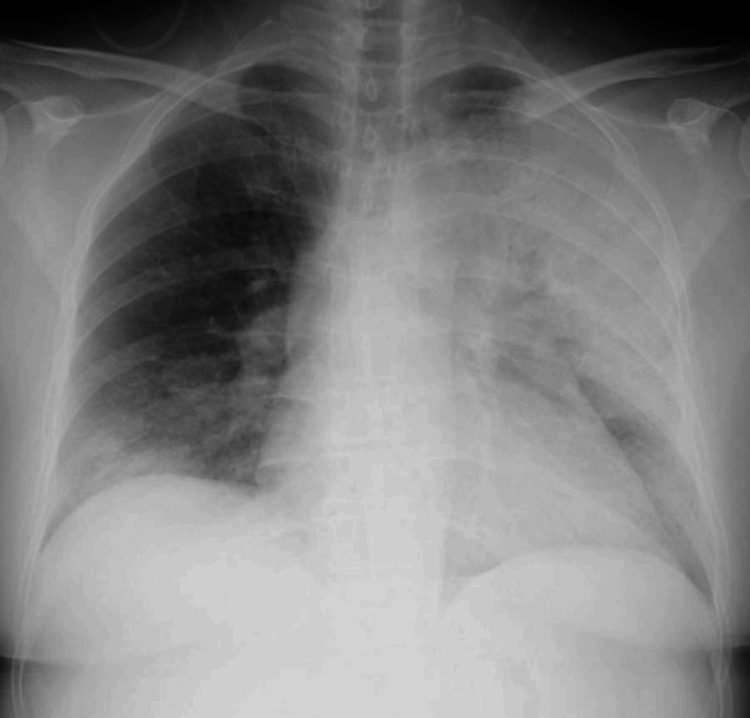
Chest X-ray on admission. Chest X-ray showed an infiltrating shadow in the left lung field.

**Figure 2:**
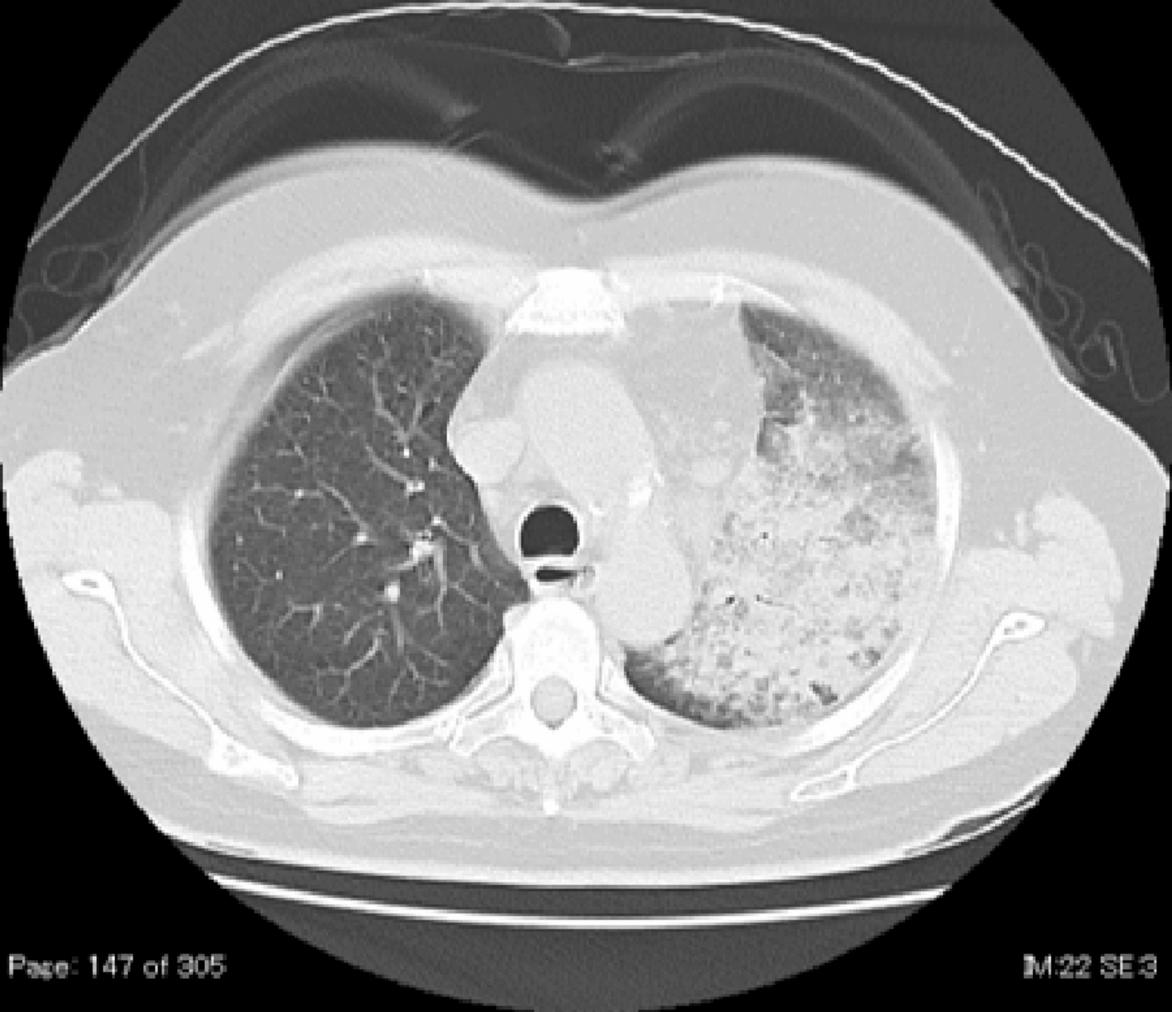
Plain CT of the chest on admission. Plain CT revealed an infiltrating shadow in the left lung.

**Figure 3:**
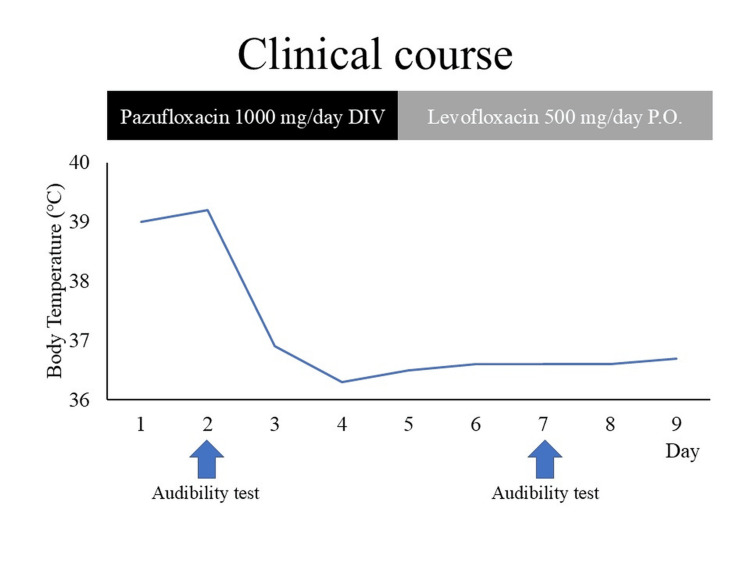
Clinical course. DIV: Intravenous drip; P.O.: Per os (by mouth).

Chest X-rays and simple CT revealed an infiltrating shadow over the entire left lung (Figures 1-2). A urine antigen test yielded a positive result for the presence of Legionella antigen, and the patient was diagnosed with a L. pneumophila infection and admitted to the Department of General Medicine on the same day. Prior to the onset of illness, the patient had neither used swimming pools, bathing facilities, nor humidifiers, nor did she have any exposure to rivers or soil, which could be considered possible infection routes. The clinical course after admission is shown in Figure 3. Antimicrobial therapy with IV pazufloxacin was initiated. By the third hospital day, the patient's fever had resolved, and her coughing symptoms were mild. Blood samples showed worsening liver dysfunction and abdominal ultrasonography showed no significant findings, so the patient was switched to oral levofloxacin on day 5 on suspicion of drug-induced liver dysfunction caused by pazufloxacin.

**Figure 4:**
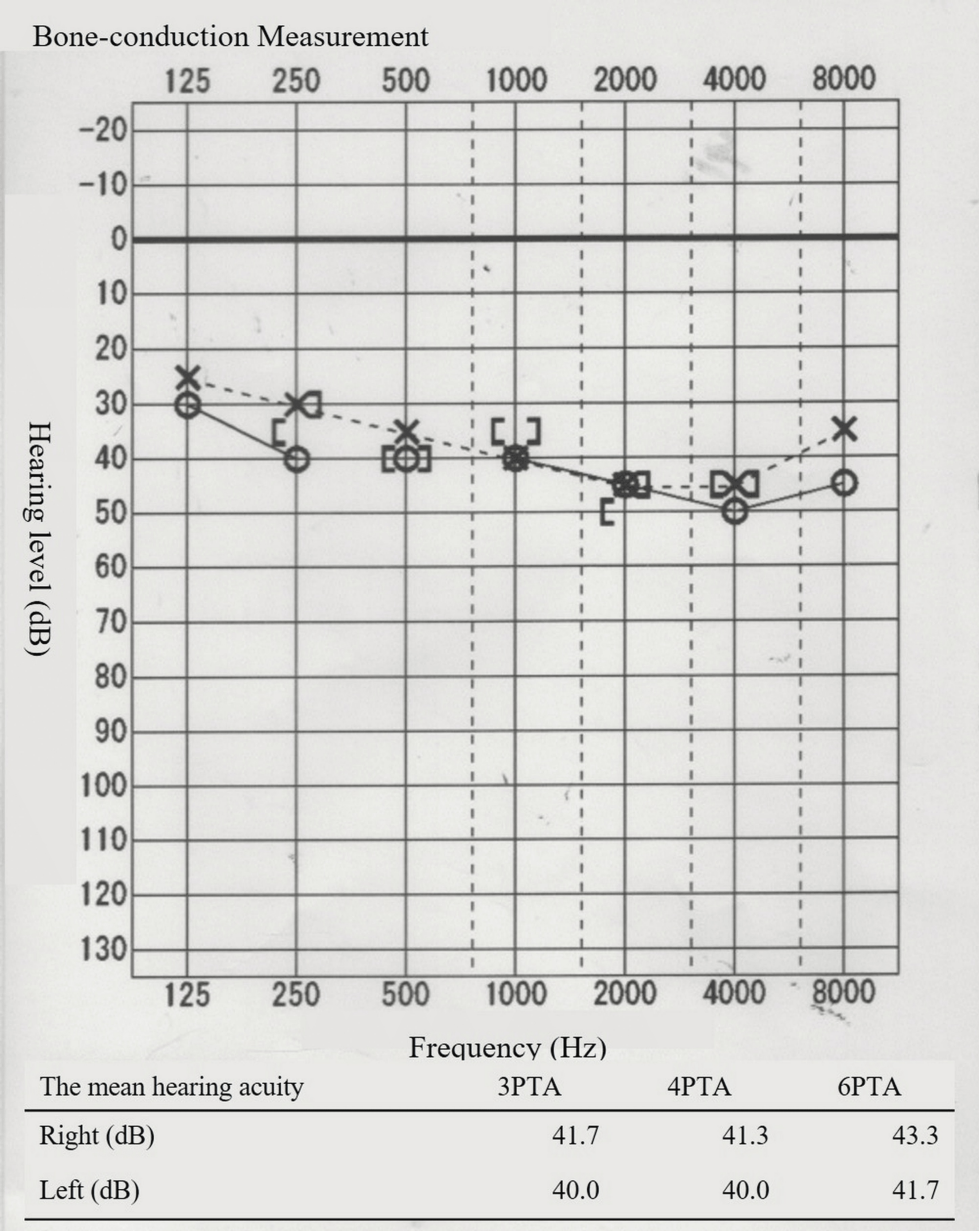
Hearing test on the second day of hospitalization. The vertical axis indicates the hearing level (dB), and the horizontal axis indicates the frequency (Hz). The patient exhibited moderate bilateral sensorineural hearing loss. The four-frequency pure tone average (4PTA) was 41.3 dB in the right ear and 40.0 dB in the left ear.

**Figure 5:**
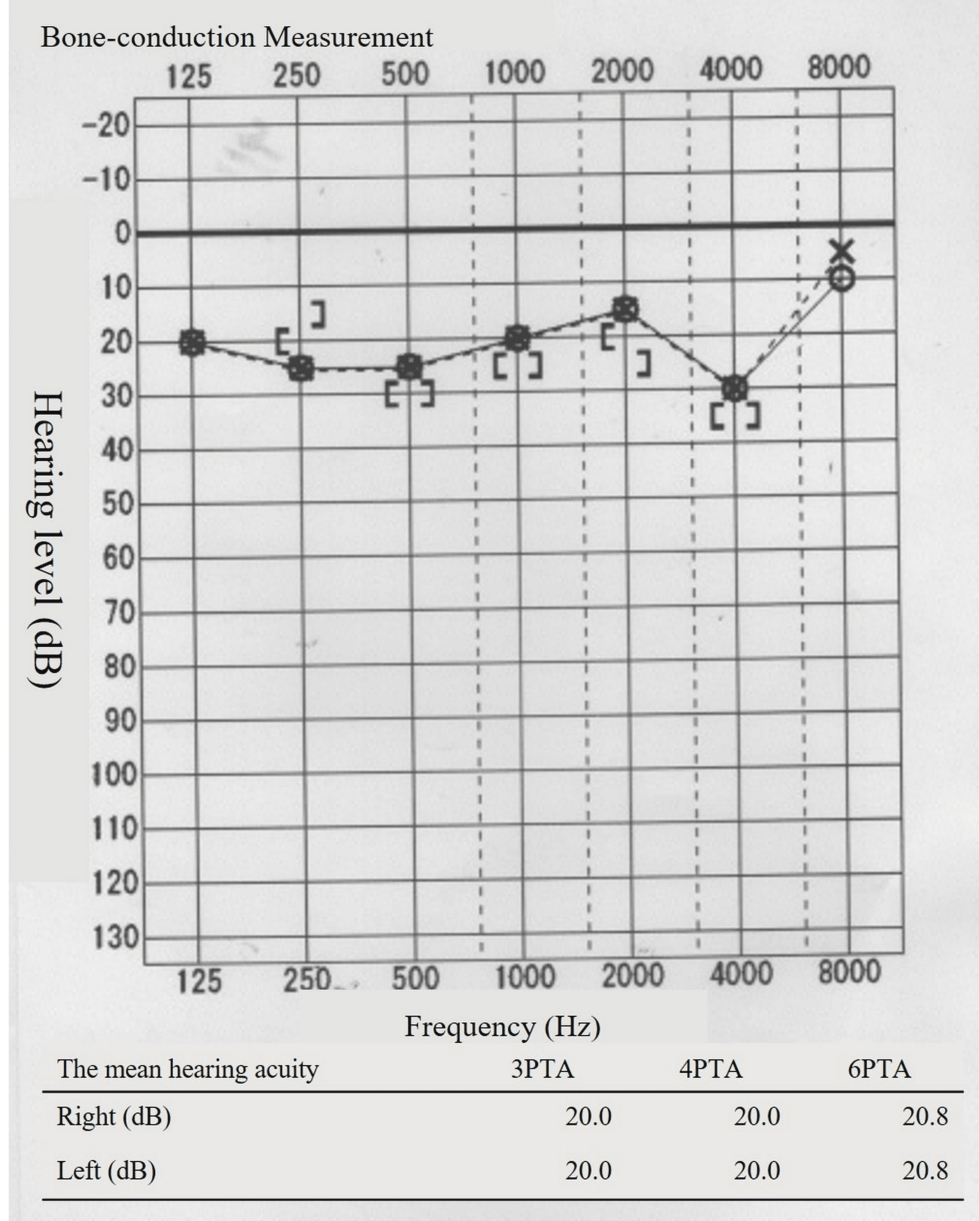
Hearing test on the seventh day of hospitalization. The vertical axis represents the hearing level (dB), and the horizontal axis represents the frequency (Hz). The bilateral sensorineural hearing loss improved significantly. The four-frequency pure tone average (4PTA) was 20.0 dB in both the right and left ears.

Regarding the patient’s hearing, she visited an otolaryngologist on the second day of hospitalization because her hearing loss persisted after admission. No abnormal findings were observed in the tympanic membrane. An audiogram revealed bilateral sensorineural hearing loss (Figure 4). As the patient was suspected to be in the early stages of age-related changes and had no difficulty in daily conversations, no new prescription was given, and the patient was followed up. As her pneumonia symptoms decreased, her hearing loss symptoms also decreased. Figure 5 shows the hearing test results performed on the seventh day. Based on the clinical course, it was inferred that the hearing loss symptoms were due to a Legionella infection. The patient was discharged on the ninth day and continued to receive antimicrobial therapy with oral levofloxacin for nine days after discharge.

## Discussion

The patient developed pneumonia, characterized by fever and dry cough, and was diagnosed with a *L. pneumophila* infection using a combination of imaging and urinary antigen tests. The pneumonia showed improvement with the administration of intravenous pazufloxacin and oral levofloxacin. The risk factors for Legionella infection include male sex, age over 50 years, smoking, diabetes, cancer, and underlying immunosuppressive conditions [1]; however, in this case, the patient was only a smoker. Legionella is primarily transmitted through the inhalation of Legionella-contaminated aerosols from artificial water sources such as air conditioning units, showers, and bathrooms [1, 5, 6]. It has also been previously isolated from laundry tubs [7], and given that the patient worked as a laundry worker, the workplace possibly served as the source of infection [8].

The patient was diagnosed through urinary antigen testing, which has a sensitivity of 74-87% and specificity of 94.7-98.3% [2].

In this case, bilateral hearing loss developed after the onset of fever and dry cough. The hearing loss persisted after admission, and a hearing test revealed bilateral sensorineural hearing loss. A PubMed search for 'Legionella' and 'Hearing Loss' revealed only one case of hearing loss associated with Legionella infection from 2002 to 2022.

Nolte JE et al. reported the case of a 64-year-old man who presented to the ED with fever, dry cough, and hearing loss and was diagnosed with Legionella infection by urinary antigen, complicated by moderate to severe sensorineural hearing loss. He was treated with levofloxacin for pneumonia, which resolved his symptoms. In the reported case, sudden hearing loss was suspected and treated with oral prednisolone (60 mg/day), and hearing improved [4].

Initially, an audiogram in our case showed bilateral sensorineural hearing loss with a high-frequency domain predominance, which, together with the pattern of hearing loss and age, was considered age-related hearing loss. Because the hearing loss did not interfere with daily conversation, the patient was followed up without aggressive therapeutic intervention. Antimicrobial therapy with pazufloxacin and levofloxacin improved her pneumonia symptoms and hearing loss, which suggested that the hearing loss may have been due to Legionella infection. Although there have been very few reports of hearing loss caused by Legionella pneumonia, if hearing loss due to Legionella pneumonia is reversible as in this case, it is possible that such cases have been underreported in the past.

Hearing loss symptoms in mycoplasma infections have been previously reported [8], suggesting that atypical pneumonia, other than Legionella, may also cause hearing loss. Otitis media is commonly observed in Mycoplasma infections [9], with tympanic membrane findings suggesting a potential association with hearing loss. The precise mechanisms underlying the neurological manifestations associated with Legionella infections remain unclear. However, post-infection brain single-photon emission computed tomography (SPECT) imaging has shown decreased cerebral blood flow, pointing to the possibility of vascular injury in Legionella infections [10]. Moreover, autopsy studies of patients with neurological symptoms related to Legionella infection failed to detect Legionella species [11], while other reports have demonstrated that *L. pneumophila* protease can induce pulmonary damage even in the absence of viable bacterial cells [12]. These observations lend support to the hypothesis that neurotoxin-like substances or immune-mediated mechanisms could play a role in the pathogenesis of neurological symptoms in Legionella infections [4]. Further research is needed to clarify the exact mechanism linking Legionella pneumonia to hearing loss.

It is possible that the hearing loss was caused by complications from other diseases, including sudden sensorineural hearing loss. However, no investigations were conducted to assess for other infections such as Mycoplasma, mumps virus, or herpes virus through serum antibody titers or imaging tests. Therefore, we cannot entirely exclude the possibility that the hearing loss may have been due to other underlying conditions or complications. Nonetheless, there is evidence suggesting that neurological symptoms in Legionella infections generally resolve as the patient clinically improves [13]. In this case, the resolution of both hearing loss and audiometric findings, alongside the improvement in respiratory symptoms, suggests that the hearing loss may have improved in response to appropriate treatment for Legionella infection.

## Conclusions

In this study, we encountered a case of bilateral sensorineural hearing loss associated with Legionella infection, where the hearing loss improved after therapeutic intervention for the infection. The clinical course suggests a possible association between Legionella infection and hearing loss.

Legionella infection should be considered as a potential cause of hearing loss in patients with pneumonia. However, due to the scarcity of reports, further studies are needed to determine the mechanisms underpinning hearing loss associated with Legionella infections and to develop appropriate treatment methods.
